# Complex Surgical Management of Permanent Patellar Dislocation in an Adolescent: An Eight-Year Follow-Up Case Report

**DOI:** 10.3390/children12121594

**Published:** 2025-11-24

**Authors:** Janina Wurster, Elias Ammann, Erich Rutz, Victor Valderrabano, Carlo Camathias

**Affiliations:** 1 Medical Faculty, University of Zurich, CH-8032 Zurich, Switzerland; 2 Department of Orthopaedic Surgery and Traumatology, Kantonsspital Baselland, CH-4101 Bruderholz, Switzerland; 3 Medical Faculty, University of Basel, CH-4056 Basel, Switzerland; erich.rutz@rch.org.au (E.R.);; 4 Department of Paediatric Orthopaedic Surgery, The Royal Children’s Hospital Parkville, Melbourne 3052, Australia; 5 Swiss Ortho Center, Swiss Medical Network, Schmerzklinik Basel, Hirschgässlein 15, CH-4010 Basel, Switzerland; 6 Praxis Momentum, Brauerstrasse 95, CH-9016 St. Gallen, Switzerland

**Keywords:** permanent patellar dislocation, trochlear dysplasia, MPFL reconstruction, femoral osteotomy, tibial osteotomy

## Abstract

**Highlights:**

**What are the main findings?**

- Comprehensive multi-level surgery achieved excellent eight-year outcomes (IKDC 10.3% → 75.9%) in adolescent permanent patellar dislocation with multiple bone deformities.
- Permanent patellar dislocation causes cascading skeletal deformities (femoral valgus, tibial varus, increased slope, 90° tubercle rotation) through chronic abnormal loading during growth.

**What are the implication of the main finding?**

- Isolated procedures fail—systematic correction of all factors simultaneously is essential for permanent patellar dislocation in adolescents.
- The proposed surgical algorithm guides clinicians in managing these complex cases and emphasises early intervention to prevent progressive deformations.

**Abstract:**

**Introduction:** This case report presents the multifactorial surgical management and long-term outcome of a permanent patellar dislocation in a 16-year-old female patient. **Case presentation:** A 16-year-old female with permanent patellar dislocation of the left knee complained of progressive pain and functional limitations. Physical examination revealed a 20-degree passive flexion contracture, significant muscle weakness in knee extension, and a permanently laterally dislocated patella that could not be manually repositioned. Imaging studies revealed a complex knee deformity characterised by femoral valgus, tibial varus, posterior tibial slope, and trochlear dysplasia. The patient underwent a comprehensive surgical approach addressing all deformities, including femoral and tibial osteotomies, trochleoplasty, medial patellofemoral ligament (MPFL) plasty, and quadriceps muscle balancing. **Results:** At the eight-year follow-up, the patient demonstrated a full range of motion and adequate stability, and experienced mild pain only rarely. Outcomes improved significantly: the International Knee Documentation Committee (IKDC) score increased from 10.3% to 75.9%, the Lysholm score rose from 24 to 72, the Kujala score improved from 22 to 67, and the BPII score increased from 14.9 to 55.4. **Conclusions:** Comprehensive surgical correction of all predisposing factors achieved sustained functional improvement over eight years, demonstrating that systematic treatment of anatomical and functional abnormalities is essential for the successful management of permanent patellar dislocation. Level of evidence: V.

## 1. Introduction

Permanent patellar dislocation, defined as persistent lateral displacement of the patella throughout the entire range of motion, is a rare condition with an unknown prevalence. Over the past three decades, the English literature has presented only case reports or small case series. This highlights the limited evidence base for treatment strategies [1,2,3,4]. Despite various surgical techniques for managing patellar instability, there remains a significant knowledge gap regarding the optimal approach for permanent patellar dislocation with multiple coexisting deformities, particularly in skeletally immature patients. Furthermore, long-term outcomes beyond 5 years are rarely reported [1,2,3,4]. Managing permanent patellar instability presents unique challenges due to its multifactorial aetiology, requiring careful consideration of various conservative and surgical treatment options [3,4,5,6]. This case report addresses these gaps by presenting comprehensive surgical management with detailed biomechanical rationale and eight-year follow-up data [7,8,9,10].

## 2. Case Report

A 16-year-old female, otherwise healthy, presented with a permanent patellar dislocation of the left knee with progressive knee pain and functional limitations. She fell frequently and could not climb stairs with her left leg or stand on this leg alone. Medical history revealed that recurrent patellar instability was initially diagnosed in early childhood but not treated. Before the age of 16, no knee treatment was performed. She did not report any major accident involving her knee.

The patient walked without limping and had a flexible flatfoot deformity, increased femoral anteversion of 45° in the left leg, and a 20-degree passive flexion contracture in the left knee. The patella was permanently dislocated laterally and could not be repositioned manually. Furthermore, a significant 2/5 muscle weakness was noted in knee extension, as assessed by the modified Medical Research Council (MRC) scale [11]. However, no neurological deficit could be detected. The knee was anteromedially and posterolaterally rotationally unstable.

**Figure 1:**
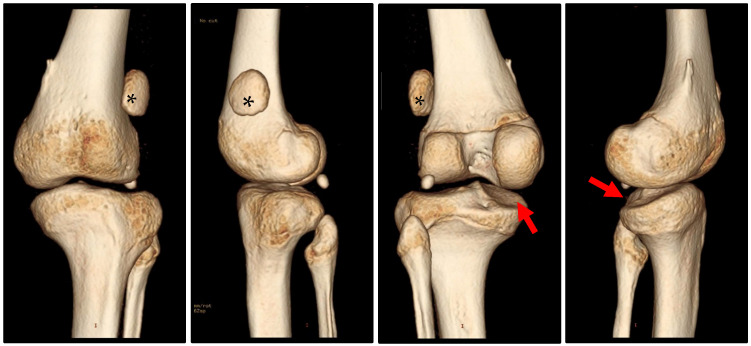
Three-dimensional CT reconstruction showing the laterally dislocated patella (*) and the complex knee deformity with a posterior tibial slope of the medial tibial plateau (arrow).

**Figure 2:**
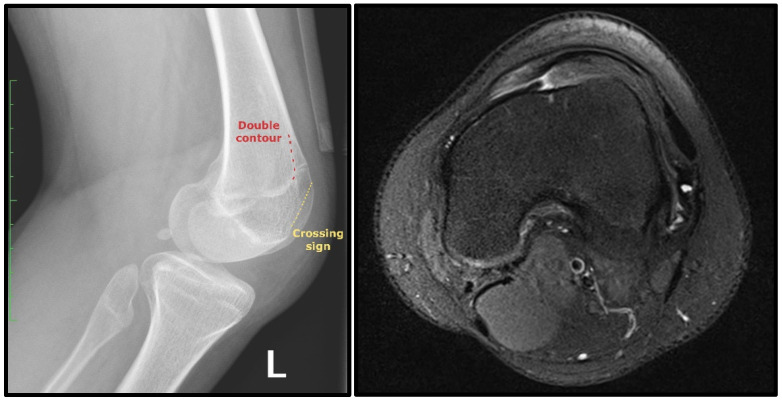
Lateral knee radiograph and axial MRI showing signs of trochlear dysplasia consistent with Dejour type B trochlear dysplasia. Radiograph with crossing sign and double contour. MRI shows a hypoplastic medial facet, whereas the lateral aspect of the trochlea appears bulged.

X-rays and Magnetic Resonance Imaging (MRI) confirmed permanent patellar dislocation and a complex deformity in the knee joint, i.e., valgus deformity of the distal femur, varus deformity of the proximal tibia, and a posterior tibial slope of the medial tibial plateau (hemislope). The bony configuration was further investigated using a 3D Computed Tomography reconstruction (3D-CT) (Figure 1). Mechanical axis alignment was assessed using standardised weight-bearing long-leg radiographs. Measurements included mechanical lateral distal femoral angle (mLDFA, normal range 85–90°), medial proximal tibial angle (MPTA, normal range 85–90°), and posterior tibial slope (PTS, normal range 5–10°). In this patient, preoperative values were mLDFA 79° (indicating valgus), MPTA 75° (indicating varus), and medial PTS 18° (significantly increased posterior slope). However, the mechanical axis did not deviate laterally. In addition, the images revealed various pathologies predictive of patellar instability, particularly trochlear dysplasia consistent with Dejour type B dysplasia, including a crossing sign and a double contour (Figure 2) [12]. MRI also indicated lateral and medial meniscus lesions and a partial rupture of the anterior cruciate ligament.

**Figure 3:**
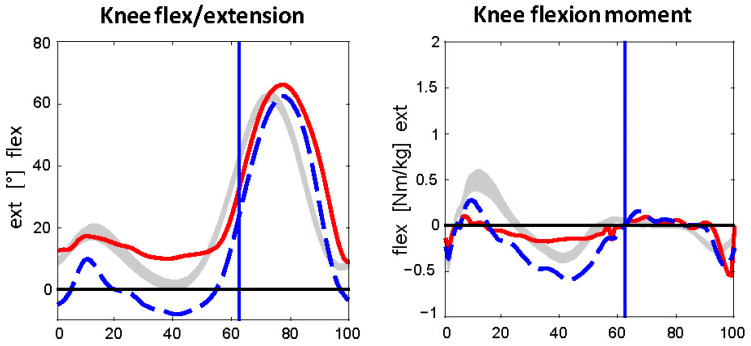
Kinematics (flexion/extension angles) and kinetics (flexion/extension moments). The red line represents the left leg; the blue line represents the right leg. During the mid-stance phase, the red line appears flat, showing that there is neither an extension in the knee joint nor sufficient muscle moments in the thigh.

Three-dimensional gait analysis was performed using a 12-camera motion capture system (Vicon Motion Systems Ltd., Oxford, UK) with a sampling rate of 200 Hz and two force plates (Kistler, Instrumente AG, Winterthur, Switzerland) at a sampling rate of 1000 Hz. The patient walked barefoot at a self-selected speed. A standard body marker set was used, and kinematic and kinetic parameters were calculated according to established protocols [13]. Gait analysis was performed to understand the compensation mechanisms: During the mid-stance phase, neither an extension in the knee joint nor sufficient muscle moments in the thigh were detectable (Figure 3). These findings were consistent with the decreased muscle strength observed during the clinical examination.

Diagnostic knee arthroscopy confirmed the medial meniscal tear and a hypermobile lateral meniscus, which was stabilised with three outside-in sutures three months before the primary surgical intervention.

Also worth mentioning is the right femoro-patellar joint, which, years later, also developed recurrent patellar instability, although it was not permanently dislocated.

## 3. Surgical Technique

Considering the patient’s severe functional limitations (inability to climb stairs, frequent falls, inability to stand on the affected leg), progressive pain, 20° flexion contracture, significant muscle weakness (MRC grade 2/5), and the poor long-term prognosis of conservative treatment with inevitable early osteoarthritis and potential knee replacement by early adulthood, comprehensive surgical correction was indicated.

Various factors had to be taken into account, as detailed below, which individually impaired the function of the knee joint but also negatively influenced one another.

Each surgical intervention was selected based on specific biomechanical considerations: (1) Femoral osteotomy corrected the valgus deformity and rotation, thereby reducing lateral patellar vector forces [14] and decreasing the TT-TG distance [15]; (2) Tibial osteotomy addressed the varus deformity and abnormal posterior slope, normalising the Q-angle and tibiofemoral mechanics [16]; (3) Trochleoplasty created an appropriate groove for patellar tracking further to improve the stability and kinematics of the patella [17,18]; (4) MPFL reconstruction restored the primary soft tissue restraint against lateral patellar translation, providing the restraining force in extension [19]; (5) Quadriceps balancing was essential to redistribute tension between medial and lateral components, counteracting the chronic shortening during development [20,21].

**Figure 4:**
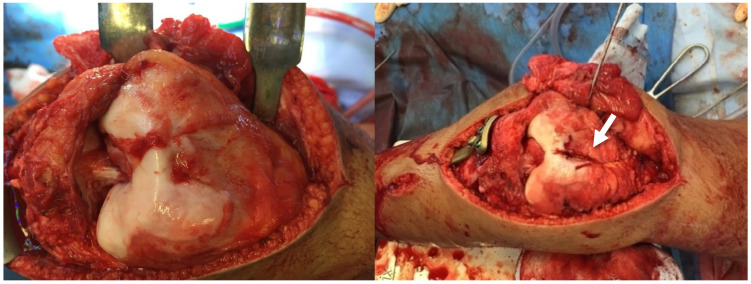
Trochlea before and after trochleoplasty with the new trochlear groove (arrow).

**Figure 5:**
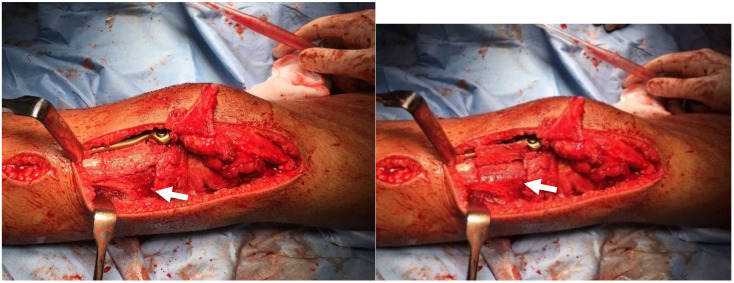
Lateral position where the tibial tubercle was detached (arrow). The tibial tubercle originated explicitly on the lateral side, 90° rotated from its considered correct location. Removal of bone for the tibial tubercle transfer located directly anterior.

The procedure involved a lateral release and detachment of the tibial tubercle to mobilise the patella. Femoral and tibial osteotomies realigned the limb, and correction of the posterior tibial slope of the medial tibial plateau to reduce overload at the ventral tibial plateau and ensure the mechanical axis continues to pass through the centre of the knee. Therefore, only the medial half of the tibial plateau was osteotomised, and the correction of both the varus and the increased slope was explicitly targeted to the medial side. The correction made to the femur and tibia was secured using plate fixation. Then, a trochleoplasty was performed using the Bereiter technique described by Camathias et al. [22], which involves creating a new groove by removing subchondral bone while preserving the articular cartilage. The bone wedge from trochleoplasty was used to lengthen the lateral trochlear facet to stabilise the patella further [22]. The Medial Patello Femoral Ligament (MPFL) -reconstruction utilised a quadriceps autograft with femoral fixation at Schöttle’s point. The former tibial tubercle osteotomy was fixed with two screws anteriorly on the tibia, approximately 90° rotated from its origin on the lateral side (Figure 4 and Figure 5).

Permanent dislocation resulted in the shortening of the quadriceps muscle. Therefore, lengthening the quadriceps tendon by balancing the four parts of the muscle was indispensable to reduce lateral traction of the muscles. Additionally, the medial vastus was transferred distally and the lateral vastus proximally to increase the tension medially and reduce it laterally. To prevent medial tilting of the patella, the lateral gap was closed using the fascia lata [9,22].

## 4. Postoperative Management

Thrombosis prophylaxis consisted of low-molecular-weight heparin (Enoxaparin 40 mg subcutaneously once daily) for 6 weeks postoperatively. The physiotherapy protocol included three phases: (1) Early phase (weeks 1–6): isometric quadriceps exercises, gentle passive range of motion within orthosis limitations of 40° flexion, and neuromuscular electrical stimulation; (2) Intermediate phase (weeks 7–12): progressive weight-bearing, closed-chain exercises, and proprioceptive training; (3) Advanced phase (months 4–9): strengthening exercises, functional training, and moderate sport-specific rehabilitation.

## 5. Results

**Figure 6:**
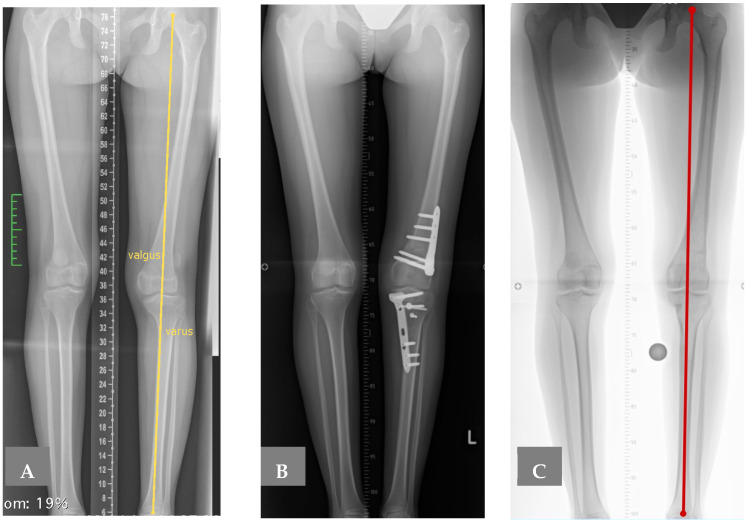
Anteroposterior radiographs showing (**A**) valgus deformity of the distal femur and varus deformity of the proximal tibia before surgery, (**B**) osteotomy plates and screws eight weeks after surgery, and (**C**) straight femur and tibia nine months after osteosynthetic material was removed.

The postoperative course was free of complications. The patient experienced minimal pain, and full weight-bearing was possible 12 weeks after surgery. Correction of the knee deformity resulted in full extension and a centred mechanical axis (Figure 6). Only hypotrophy of the quadriceps muscle and the associated active extension deficit persisted for several months despite intensive physiotherapy.

Eleven months after the initial surgery, the osteosynthetic material was removed. Six months after removal, an asymptomatic, stable knee joint with a mild active extension deficit of 5° was observed. Therefore, the patient continued physiotherapy to strengthen the extensor muscles.

A follow-up examination, conducted 3.5 years after the initial surgery, and an interview, eight years after the initial surgery, revealed a satisfied patient with adequate stability, a full range of motion, no active extension deficit, and rarely experienced mild pain. Table 1 presents scores measured before and after the patient’s surgery. Preoperative scores revealed markedly diminished performance, which improved significantly following surgical intervention. However, compared to patients undergoing stabilisation for recurrent patellar dislocation alone, this patient’s values remain approximately 10–20% lower, reflecting the complexity of the case.

## 6. Discussion

The causes of a permanent patellar dislocation are unclear, as this condition is relatively rare and complex. However, a permanent patellar dislocation is a complicated condition that can be seen as a systemic failure, where the constant lateral pull of the knee extensor apparatus leads to the deformation of various structures in the knee. This condition is particularly impactful during the growth period, when the growth plates play a crucial role. The ongoing lateral tension and misalignment caused by the dislocation influence the development of these growth plates, potentially leading to asymmetric or abnormal growth patterns. The progressive bony adaptations observed in this case align with the concept of Hueter and Volkmann, whereby bone remodels in response to mechanical loading [27,28]. As the body undergoes these changes, there is an inherent attempt to compensate and counteract the misalignment, striving to maintain a straight axis. However, this compensatory mechanism often results in additional deformations at other sites. In the case studied, the femur developed an increased distal valgus deformity, resulting in a compensatory varus deformity in the proximal tibia. However, overall, this resulted in a straight-leg axis. The body’s adaptation to the unnatural forces exerted by the dislocated patella can create a cascading effect of imbalances and structural alterations, not only in the knee but potentially affecting the overall posture and biomechanics of the individual. This highlights the importance of early intervention and holistic management in cases of permanent patellar dislocation, addressing both the primary issue and its systemic implications. Nonetheless, this proposed mechanism of development and its corresponding speculation, while conjectural, demonstrate a consistency in the details. In this context, the risk factors for patellar dislocation should be considered individually and critically: The distal femur’s trochlea is the patella’s primary static stabiliser [18]. Hence, trochlear dysplasia, defined as a bony abnormality of the trochlea with a flat or even convex trochlear groove and asymmetric condyles, is one of the main risk factors for patellar instability and has been identified in 96% of patients with patellofemoral dislocation [29]. Therefore, in adolescents and young adults with severe trochlear dysplasia (Dejour type B or worse), trochleoplasty has demonstrated excellent outcomes with minimal complications at a minimum 2-year follow-up [30,31]. However, when a permanent patellar dislocation occurs in the early years, there is a risk that the trochlea may not develop properly. This patient, who had had a permanent patellar dislocation since early childhood, exhibited only a rudimentary trochlea with minimal cartilage lining. It is probable that the pressure exerted by the patella and the natural bending motion, crucial for developing a functional trochlea, were lacking in the patient. (Figure 4)

However, patellar instability has a broad multifactorial aetiology that further includes the lateral position of the tibial tubercle. An increased Tibial Tubercle—Trochlear Groove distance (TT-TG distance) is a significant predisposing factor for patellar instability, trochlear dysplasia and patella alta [7,32,33]. However, recent studies question the validity of an increased TT-TG distance for determining a laterally positioned tibial tuberosity, as many other factors, such as the degree of knee flexion, rotational instability of the knee joint, and ligamentous laxity, influence this value [34,35]. Nonetheless, in this case, a tibial tubercle was positioned far laterally, almost rotated by 90 degrees around the tibia. The extreme lateral position of the tibial tubercle may result from chronic laterally directed force vectors of the quadriceps during growth. Moreover, this case illustrates that a significantly laterally displaced tibial tubercle can hinder or compromise the stability of the femoropatellar joint. However, the underlying causes of such displacement remain elusive. It appears to be associated with a complex interplay of various static and functional factors, which are yet to be fully understood.

In the same vein, considerations that include the importance of coronal and rotational limb alignment are relevant [4]. Especially with knee valgus alignment, the lateral displacement force is increased, thereby increasing the risk of lateral patellar dislocation [4,36]. A recent systematic review has demonstrated that distal femoral osteotomies significantly improve recurrent patellar instability in patients with genu valgum deformity [37]. Whether the femur’s valgus or the tibia’s varus occurred first is questionable. However, the additional increase in tibial slope led to an extension deficit, which could plausibly have arisen from the laterally and dorsally directed force. Studies have shown that asymmetric tibial slope, particularly increased medial posterior slope, significantly influences femoral rotation and contributes to lateral patellar instability through altered biomechanics [38].

In this patient, various co-factors may have collectively contributed to the permanent dislocation of the patella. These include generalised hypermobility, skeletal immaturity, and weakness in the quadriceps, particularly in the Vastus Medialis Obliquus (VMO) muscle. Additionally, a positive family history, the presence of a flexible flatfoot deformity, and likely rotational instability of the knee were also contributing factors. Each of these elements, when combined, could have contributed to the permanent patellar instability observed [33,39,40].

This case should also draw attention to the possible relationship between patellofemoral instability and posterolateral rotatory instability of the knee. Approaches that could explain the underlying biomechanics were provided by Camathias, arguing that an increased tibiofemoral rotation due to insufficient meniscal attachment, i.e., hypermobile lateral meniscus, may contribute to more external rotation of the tibia [9,41,42]. This, in turn, leads to increased TT-TG distance, affecting patellofemoral stability [35]. A study by Prakash et al. supports these approaches, as they showed that the cause of increased knee rotation in patients with patellar instability is at the level of the knee joint itself [34].

Reflecting the multifactorial aetiology, the repertoire of surgical interventions is vast, and a universal therapeutic approach to treating patellar instability cannot be defined [3,4]. This case, however, illustrates that comprehensive diagnostics and evaluation of all predisposing factors are a critical basis for surgical considerations.

In principle, the surgical procedure should be expanded as the severity of patellar instability increases. Thus, isolated MPFL reconstruction can be sufficient for patients with recurrent patellar instability [6]. However, appropriate surgical interventions must be supplemented if additional risk factors are present. Recent evidence demonstrates that double-level knee derotational osteotomy combined with MPFL reconstruction yields superior postoperative outcomes compared to isolated soft tissue procedures in patients with severe malrotation [43]. In cases of permanent patellar dislocation, there is a scenario where an entire system has failed yet has found a new balance. Correcting individual factors alone would further disrupt this prevailing balance and could worsen the outcome. Thus, fixing only the femur would medialise the load-bearing axis, while solely correcting the tibia would lateralise it.

Moreover, shortening of the quadriceps muscle is common in permanent patellar dislocation with a laterally positioned tibial tubercle and a valgus deformity. Therefore, lengthening the quadriceps tendon is essential [44]. Otherwise, the lateral traction of the muscles would re-dislocate the patella. Thus, to achieve adequate stability, extensive surgical correction of all predisposing factors is necessary. This case demonstrates that a satisfactory long-term outcome can be achieved by diligently addressing all factors. However, it also raises the question of whether earlier intervention in childhood might have prevented such extensive bone deformations.

Our case shares significant similarities with other reported cases of permanent patellar dislocation, whilst also presenting unique features. Teküstün et al. recently reported on eight skeletally mature patients with eleven chronic, fixed, and permanent lateral patellar dislocations who underwent comprehensive, single-stage surgical correction [34]. Their approach, which they termed “all-in-one treatment,” included combined proximal and distal realignment procedures similar to our strategy. With a mean follow-up of 11.3 years, they demonstrated significant functional improvement (mean Kujala score from 40.89 to 68.3) and no instances of recurrent instability, supporting our finding that comprehensive correction of all anatomical abnormalities is essential for successful outcomes. However, their patient population consisted exclusively of skeletally mature adults. In contrast, our patient was a skeletally immature adolescent at the time of surgery, highlighting the applicability of comprehensive surgical approaches across different age groups.

Based on our experience and this eight-year follow-up result, and consistent with recent comprehensive reviews on treatment options for recurrent patellar instability in children and adolescents [45], we propose the following surgical algorithm for similar complex cases of permanent patellar dislocation in skeletally immature patients:

1. Comprehensive preoperative assessment, including MRI, 3D-CT, long-leg radiographs, and, if possible, gait analysis to identify all contributing factors and functional impairments.
2. Correction of bony malalignment through femoral and tibial osteotomies as the foundation.
3. Trochleoplasty when trochlear dysplasia is severe (Dejour type B or worse).
4. Tibial tubercle transfer.
5. MPFL reconstruction using autograft.
6. Quadriceps balancing with lengthening when extension deficit exceeds 15°.
7. Detailed rehabilitation protocol with at least 12 months of physiotherapy.

This algorithmic approach ensures that all pathological factors are systematically addressed, which our case suggests is crucial for long-term success.

## 7. Conclusions

This case report demonstrates the importance of a holistic approach to treating permanent patellar dislocation. Comprehensive diagnostics and evaluation of all predisposing factors provided the foundation for successful surgical intervention. The long-term outcome confirms that a satisfactory result can be achieved by diligently correcting all contributing anatomical and functional factors. Early intervention in childhood might prevent the development of extensive bone deformations. Surgeons should consider the complex interplay of multiple factors in these challenging cases and tailor the surgical approach accordingly.

## Figures and Tables

**Table 1:** Comparison of the results of the clinical scores before surgery, 1.5 and 3.5 years after surgery, and eight years postoperatively. (BPII: Banff Patella Instability Instrument, Kujala Score, Lysholm Score, IKDC Form) [23,24,25,26].

	BPII	Kujala Score	Lysholm Score	IKDC Form
Before surgery	symptoms/physical complaints: 30 work-related concerns: 30 recreation/sport: 5.5 lifestyle: 22 social/emotional: 5 overall: 14.9/100	22/100	24/100	9/87 (10.3%)
1.5 y follow-up	symptoms/physical complaints: 80 work-related concerns: 75.5 recreation/sport: 12.5 lifestyle: 55.5 social/emotional: 45 overall: 42.9/100	51/100	50/100	52/87 (59.8%)
3.5 y follow-up	symptoms/physical complaints: 85 work-related concerns: 88 recreation/sport: 20 lifestyle: 75 social/emotional: 72 overall: 56.2/100	60/100	72/100	61/87 (70.1%)
8 y follow-up	symptoms/physical complaints: 92 work-related concerns: 87.5 recreation/sport: 21.5 lifestyle: 73.5 social/emotional: 60 overall: 55.4/100	67/100	72/100	66/87 (75.9%)

## Data Availability

The original contributions presented in this study are included in the article. Further inquiries can be directed to the corresponding author.
